# Involvement of haptoglobin phenotypes and genotypes in non-muscle invasive bladder cancer: A possible prognostic marker for risk stratification

**DOI:** 10.17179/excli2019-1768

**Published:** 2020-03-10

**Authors:** Nazi Aghaalikhani, Mojtaba Zamani, Abdolamir Allameh, Amir Mashayekhi, Pejman Shadpour, Marzieh Mahmoodi, Nadereh Rashtchizadeh

**Affiliations:** 1 Drug Applied Research Center, Tabriz University of Medical Sciences, Tabriz, Iran; 2 Department of Biochemistry and Clinical Laboratories, Faculty of Medicine, Tabriz University of Medical Sciences, Tabriz, Iran; 3 Department of Agronomy and Plant Breeding, School of Agriculture, University of Tehran, Karaj, Iran; 4 Department of Clinical Biochemistry, Faculty of Medical Sciences, Tarbiat Modares University, Tehran, Iran; 5 Department of Immunology, School of Medicine, Dezful University of Medical Sciences, Dezful, Iran; 6 Hasheminejad Kidney Center (HKC), Hospital Management Research Center (HMRC), Iran University of Medical Sciences (IUMS), Tehran, Iran; 7 Faculty of Health and Nutrition, Bushehr University of Medical Sciences, Bushehr, Iran

**Keywords:** haptoglobin, acute-phase protein, biomarkers, non-muscle invasive bladder cancer, electrophoresis

## Abstract

The association of haptoglobin (Hp) with various cancers has been reported and also it has been documented that the Hp phenotypes/genotypes have different functional ability. So, we examined phenotypes/genotypes of Hp in newly diagnosed, untreated non-muscle invasive bladder cancer (NMIBC) patients and investigated its prognostic value for risk stratification of the cancer. In eighty NMIBC patients and 80 healthy individuals the Hp genotypes and phenotypes were analyzed using polymerase chain reaction (PCR) and two-dimensional gel electrophoresis (2D-GE), respectively. Besides, the presence of the Hpα1, α2, and β chains in the sera was confirmed by Mass Spectrometry (MS). The frequencies of the *1-1* and *2-2* genotypes/phenotypes were respectively higher and lower in healthy subjects compared to the patients. Our results revealed that the *2-2* genotype/phenotype could increase the risk of NMIBC. There was a positive association between the *2-2* genotype/phenotype with the T category/grade of cancer (p<0.05). The present study implied a strong association between the Hp phenotypes and genotypes with NMIBC. It was found that the *2-2* genotype and phenotype could be a risk factor for NMIBC incidence, as well as, progression. This study introduced Hp genotyping as a possible cost-effective and precise method for prognosis of individuals at the risk of NMIBC.

## Introduction

Bladder cancer, the fourth most prevalent cancer in men, is three times less common among women but steadily growing in the prevalence. It has been reported that the median age of diagnosis for bladder cancer is 65 years, while it occasionally occurs under 40 years of age (NCCN*, *2016[23]; Scosyrev et al., 2009[26]). Non-muscle invasive bladder cancer (NMIBC) and muscle-invasive bladder cancer (MIBC) are the two clinically identified bladder cancers. NMIBC comprises about 75 % of initially diagnosed patients. In about 90 % of NMIBC patients with early diagnosis and treatment, at least five-year survival could be expected, while in the patients who left unchecked (over 70 % cases) the recurrence rate of the cancer is very high. Up to 25 % of NMIBCs may eventually upstage and become invasive. The muscle invasiveness, along with higher grade, warrants less favorable prognosis due to disease progression and eventual metastasis (NCCN, 2016[23]; Scosyrev et al., 2009[26]; Shadpour et al., 2016[27]). Traditionally, early diagnosis of bladder cancer may entail years of scheduled monitoring with cystoscopies and occasional transurethral resections (Avritscher et al., 2006[2]). However, urine cytology, urine tests like bladder tumor antigen (BTA) and nuclear matrix protein-22 (NMP-22), and newer molecular and genetic markers that detect gene mutations such as RAS, FGFR3, PIK3CA, and TP53 in urinary sediment may help in the early detection and prediction of urothelial carcinomas (van Rhijn et al., 2005[29]). Currently, in order to diagnosis of bladder cancer, no appropriate urinary assays exist to replace urine cytology and cystoscopy, with or without biopsy (van Rhijn et al., 2005[29]).

Haptoglobin (Hp), a sialoglycoprotein produced mainly by the liver, is composed of α- and β-chains (a tetrameric protein with two α/β dimers). Hp β-chains are same in all types and the α-chains (α1 and α2) are exclusively susceptible to polymorphisms. Expression of Hp is controlled by two alleles Hp1 and Hp2, located on the chromosome 16q22 in humans and present as three major phenotypes Hp1-1, Hp2-2, and Hp1-2. Hp1-1 is stronger than other phenotypes in antioxidant properties binding to free hemoglobin. However, angiogenic potential in Hp2-2 is higher than Hp1-1 and Hp1-2. It can be said that the Hp1-2 phenotype displays moderate functional features in comparison with the other phenotypes (Langlois and Delonghe, 1996[17]; Cid et al., 1993[9]). Several studies have examined the possible association between Hp types and its phenotypes/genotypes with various cancer incidences (Carter and Worwood, 2007[7]). In this regard, increased levels of serum Hp have been reported in a variety of malignancies such as lung (Lu et al., 2016[19]), breast (Awadallah and Atoum, 2004[3]; Tabassum et al., 2012[28]), liver (Ibrahim et al., 2012[13]), kidney (Burbea et al., 2004[6]), and ovarian cancers (Mandato et al., 2012[20]).

Considering possible association between the Hp and cancers and on the other hand, different functional ability of Hp phenotypes and genotypes, in the present study we examined phenotypes/genotypes of Hp in patients with NMIBC and investigated its prognostic value for risk stratification of the cancer.

## Materials and Methods

### Patients and sample preparation

Eighty NMIBC patients and 80 healthy individuals were recruited in this study. The patients were prospectively enrolled based on their clinical diagnosis of bladder cancer at Hasheminejad Kidney Center (HKC), a national referral center for urologic patients, via pathologic results *(from March 2016 to March 2018). *Newly diagnosed primary untreated cases with NMIBC with no history of chronic illness, immunotherapy, chemotherapy or prior exposure to blood products were included in the study. All participants were informed about the purpose of the study and informed consent was obtained from all individual participants included in the study. Healthy individuals without any type of cancers and chronic disease were selected as the control group. The present study protocol was reviewed and approved by the Ethical Committee of Tabriz University of Medical Sciences [Reg. No. TBZMED.REC.1394. 315].

Fasting blood samples (5 ml) from the patient (before surgery) and control individuals were taken and were used for serum isolation and a fraction collected in an EDTA-containing tube for DNA extraction. Histopathological classification of stage and grade were performed by an experienced uropathologist in accordance with the 2004 American Joint Committee on Cancer (AJCC) TNM staging system and 2004 WHO/International Society of Urological Pathology classification systems, respectively.

### Hp phenotyping using two-dimensional gel electrophoresis (2D-GE)

#### Enrichment of serum haptoglobin

The purpose of this step was to remove non-protein contaminants (Damerval et al., 1986[10]). Serum samples of 100 µl were incubated with 1 ml cold acetone solution containing 10 % tri-chloroacetic acid (TCA) and 20 mM dithiothreitol (DTT) at -20 °C for 12 hours. Then, the mixture was centrifuged at 17000 g for 15 minutes at 4 °C. The supernatant was discarded and the pellets incubated with 1 ml cold acetone solution (containing 20 mM DTT) at -20 °C for an hour. The mixture was then centrifuged at 12000 g for 15 minutes at 4 °C. The pellets were re-suspended in a lysis buffer composed of 7M Urea, 2M Thiourea, 4 % (w/v) 3-[(3-cholamidopropyl) dimethyl ammonio]-1-propane sulfonate (CHAPS), IPG buffer (pH=4-7) and 35 mM Tris at room temperature. 

#### Serum protein quantification

To achieve a uniform loading for two-dimensional gel electrophoresis (2D-GE), the protein concentration of the dissolved samples in rehydration buffer were determined using the Bradford method (Bradford, 1976[5]).

#### Rehydration of IPG strips

150 µg of the proteins was diluted to 320 µl with rehydration buffer (8 M Urea, 2 % CHAPS, 50 mM DTT, 0.2 % IPG buffer at pH=4-7) and 0.002 % Bromophenol Blue. The IPG strips (Bio-Rad; 18 cm; pH=4-7) were rehydrated for 11-16 hours.

#### First dimensional analysis by isoelectric focusing (Using Multiphor II; Bioscience Amersham)

Isoelectric focusing (IEF) run was done using the following protocol: 150 vh between 0 - 300 v, 300 vh between 300 - 500 v, 2000 vh between 500 - 3500 v and in fourth step, 39500 vh in 3500 v.

#### Second dimensional analysis by SDS-PAGE (using Protean II Xi Cell)

After IEF, the IPG strips were equilibrated in two different buffers (for 20 minutes in each equilibration buffer). The first and second equilibration buffers were composed of 6M Urea, 0.375 M Tris-Hcl (pH=8.8), 20 % Glycerol, 2 % SDS, and 2 % DTT and 2.5 % Iodoacetamide respectively. Then the proteins were analyzed using SDS-PAGE (sodium dodecyl sulfate polyacrylamide gel electrophoresis) and stained with silver nitrate. Protein spots from the stained gel with silver nitrate were removed and investigated by Mass Spectrometry.

### Hp genotyping using and polymerase chain reaction (PCR)

Genomic DNA was extracted from the peripheral blood mononuclear cells by DNA extraction kit (Genomic DNA Blood/Culture Cell Mini Kit; FavorGen). For amplification of the specific sequences of the *HP1* allele (1757-bp) and *HP2* allele (3481-bp), primers A (5-GAGGGGAGCTTGCCTTTCCATTG-3′) and B (5′-GAGATTTTTGAGCCCTGGCTGGT-3′) and for amplification the specific sequence of the *HP2* allele (349-bp), primers C (5′-CCTGCCTCGTATTAACTGCACCAT-3′) and D (5′-CCGAGTGCTCCACATAGCCATGT-3′) were used, respectively (Koch et al., 2002[16]; Li et al., 2009[18]). PCR was performed in a final volume of 20 μl comprising 100 ng of genomic DNA. For genotyping Hp two protocols were used as follows:

Protocol 1) 1×PCR buffer (Ampliqon, Odense, Denmark), 0.2 mM dNTPs, 2 mM MgCl_2_, 1 μM of each primer (A and B) and 2 units of Ampliqon taq DNA polymerase were used. Amplification conditions used with thermal cycler were: a primary incubation for 10 minutes at 95 °C followed by 40 cycles of incubation at 95 °C for 30 seconds, 35 seconds at 68 °C, and 120 seconds at 72 °C, with a final extension 10 minutes at 72 °C.

Protocol 2) 1×PCR buffer (Ampliqon), 0.1 mM dNTPs, 2 mM MgCl_2_, 1 μM of each primer (C and D) and 2 units of Ampliqon taq DNA polymerase were used. Amplification conditions used with thermal cycler were: a primary incubation for 10 minutes at 95 °C followed by 40 cycles of incubation at 95 °C for 30 seconds, 30 seconds at 69 °C, and 30 seconds at 72 °C with a final expansion 10 minutes at 72 °C.

First, protocol 1 was performed on blood samples with primers A and B. Then, in the presence of the 1757-bp product, protocol 2 was performed with primers C and D. Finally we ran the obtained PCR products on 1.5 % agarose gel electrophoresis.

### Statistical analysis

Statistical analysis was done using the Statistical Package for Social Sciences (SPSS version 16.0). Chi^2^ test helped evaluate the distribution of Hp phenotypes/ genotypes among patients and healthy controls (when a cell with n<5 was present, Fisher's exact test was used). The cutoff for the level of statistical significance was defined as p <0.05.

## Results

Clinical, pathological and demographic information of the participants are given in Table 1(Tab. 1). The percentage of patients with tumor in the pathological stages of Ta and T1 was 57.5 % and 42.5 % respectively and the grade of the tumors was 48.8 % low and 51.2 % high. 

**Figure 1:**
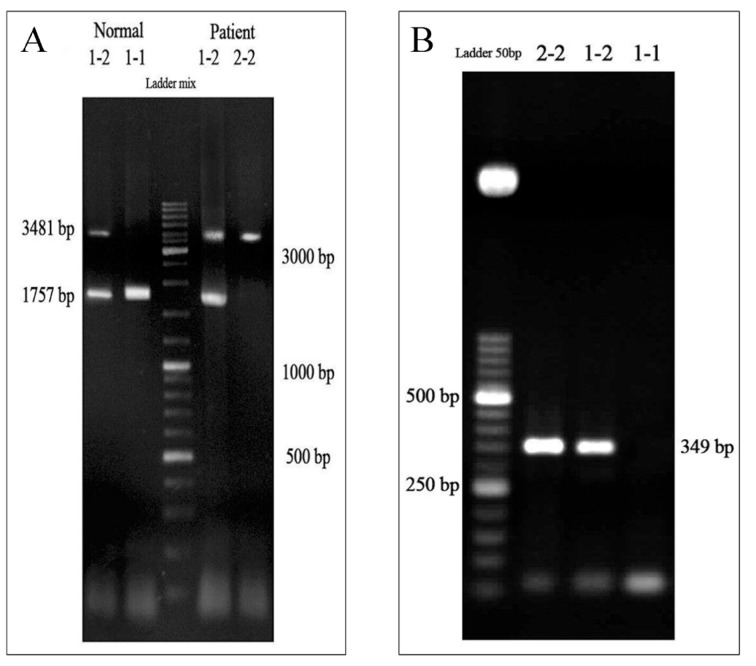
Haptoglobin (Hp) genotyping by polymerase chain reaction (PCR) (A) PCR with the protocol 1 (primers A and B) was performed and the products have been run on agarose gel and visualized under UV light. The *HP1/HP1* (*1-1*), *HP2/HP2* (*2-2*), and *HP1/HP2* (*1-2*) genotypes were characterized by single 1757-bp bands, single 3481-bp bands, and existence of the both bands, respectively. (B) PCR with the protocol 2 (primers C and D) was conducted to confirm the genotyping and observation of a 349-bp band was considered as *HP2/HP2* (*2-2*) and *HP1/HP2* (*1-2*) genotypes, whereas lack of such band implied the genotype of *HP1/HP1* (*1-1*).

The distribution of *HP* genotypes was evaluated and is shown in Figure 1A(Fig. 1). *HP1/HP1*, *HP2/HP2*, and *HP1/HP2* genotypes were characterized by single 1757-bp bands, single 3481-bp bands, and the existence of both bands, respectively. Furthermore, to confirm the genotypes, PCR with protocol 2 was conducted (Figure 1B(Fig. 1)).

Frequency of the genotypes between healthy and NMIBC groups was significantly different (p<0.001, Table 2(Tab. 2)), as we found that the frequency of the *HP1/HP1* and *HP2/HP2* genotypes were respectively higher and lower in healthy subjects compared to the patients (46.2 % vs. 18.75 % and 15 % vs. 37.5 %, respectively). Further logistic regression analysis also revealed that the *HP2/HP2* genotype could have a detrimental effect and possibly increase the risk of non-muscle invasive bladder cancer about 5 folds. Analysis of the *HP* allele's frequency showed similar results. The results showed that the *HP1* allele frequency was higher in the control group than in the cancer patients (65.6 % vs. 40.6 %), while the number of patients carrying the *HP2* allele was higher in comparison with the healthy individuals (59.4 % vs. 34.4 %, p<0.001, Table 2(Tab. 2)). Our results demonstrated that existence of the *HP2* allele could increase the risk of bladder cancer about 2.8 times.

**Figure 2:**
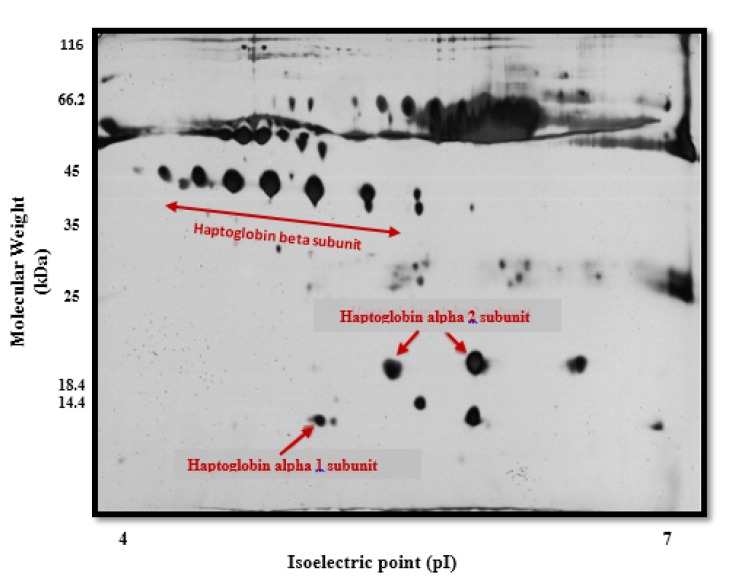
Haptoglobin (Hp) phenotyping by two-dimensional gel electrophoresis/mass spectrometry (2D-GE/MS) A total of 150 µg of serum protein from control and patient groups were analyzed by two-dimensional gel electrophoresis. Serum proteins were first separated on pI=4-7 IPG strips and then on sodium dodecyl sulfate polyacrylamide gel electrophoresis (SDS-PAGE) and stained with silver nitrate. Protein spots from the stained gel with silver nitrate were removed and investigated by mass spectrometry (MS). α1, α2 and β haptoglobin chains were observed at 13 (pI~5.2), 20 (pI~5.4-5.6) and 40-45 (pI~4.6-5.6) kDa, respectively (indicated by arrows) in control and patient serum samples.

Protein analysis of the Hp phenotypes in the serum samples using 2D-GE/mass spectrometry revealed three chains including α1 (13 kDa), α2 (20 kDa), and β (40-45 kDa) (Figure 2(Fig. 2)). As shown in Table 2(Tab. 2), the frequency of the Hp phenotypes was significantly different between healthy and patient groups (p<0.001). We observed that individuals with the Hp2-2 phenotype are probably at higher risk of developing NMIBC (about 6 folds) when compared to the patients having other Hp phenotypes. 

Our results revealed that patients with the *HP2/HP2* genotype had a higher grade (*p*=0.002, Table 3(Tab. 3)) and T1-category tumor (*p*=0.001, Table 3(Tab. 3)), while *HP1/HP2* and *HP1/HP1* carrying patients had tumors with lower grade/Ta-category. As shown in Table 3(Tab. 3), high grade/T1-category cancer was seen mostly in patients with Hp2-2 phenotype.

## Discussion

In the present study, we investigated if there is an association between Hp phenotypes and genotypes with NMIBC. Our results showed that the *HP2/HP2* genotype frequency was higher in patients with NMIBC compared to healthy individuals. We found the *HP2* allele and consequently the *HP2/HP2* genotype can be considered as a possible risk factor for NMIBC. In contrary, the *HP1/HP1* genotype showed a protective effect against the cancer. These findings revealed that functional polymorphism of Hp which can affect its antioxidant and angiogenic potentials could increase risk of the bladder cancer. So, it could be postulated that the polymorphism of Hp can be considered as a valuable prognostic marker for stratification of individuals at the risk of bladder cancer; the PCR-based analysis of Hp polymorphism seems to be a rapid and exact method for stratification. In accordance with our findings, Chen and colleagues found lower frequency of the *HP1* allele in patients with head and neck cancer compared to normal individuals (Chen et al., 2008[8]). Besides, Awadallah and Atoum (2004[3]) have documented over-displaying of the *HP2* allele in non-familial breast cancer patients. However, no study has been conducted on the association between the *HP* genotypes and bladder cancer. 

The analysis of Hp phenotypes almost supported the data obtained for genotyping, as we observed higher frequency of the Hp2-2 phenotype in the NMIBC patients in comparison with the healthy subjects. Individuals who were carrying the Hp2-2 phenotypes were at the risk of bladder cancer. In support of these findings, association between the Hp2-2 phenotype and gastric cancer has been reported (Jayanthi et al., 1989[14]). In contrary, Dimopoulos et al. (1983[12]) found no significant association between the distribution of Hp phenotypes and bladder cancer. Furthermore, low frequency of the Hp2-2 phenotype was reported in patients with bladder cancer (Benkmann et al., 1987[4]). Such controversial results could be due to technical issues in phenotyping as we used a more precise and sensitive method (2D-GE) than previous studies (starch gel electrophoresis). Besides, we just recruited untreated patients with NMIBC and not all types of bladder cancers.

Such an association between the *2-2* genotype/phenotype with NMIBC can be related to its lower antioxidant capacity and higher angiogenicity potential compared to the *1-1* genotype/phenotype (Langlois and Delonghe, 1996[17]; Cid et al., 1993[9]). In this regard, the role of hemoglobin oxidation in cancer development has been demonstrated (Angeli et al., 2011[1]; Della Rovere et al., 1995[11]) and thus the *2-2* genotype/phenotype with poor efficacy of binding to hemoglobin attenuates protection against free radicals (Langlois and Delonghe, 1996[17]). It is assumed that the *2-2* genotype/ phenotype can cause accumulation of free radicals and other noxious reactive oxygen species in the free hemoglobin (Kaplan, 2002[15]; Melamed-Frank et al., 2001[21]) and so facilitates cancer formation. On the other hand, high potential of the Hp2-2 in angiogenesis (Langlois and Delonghe, 1996[17]; Cid et al., 1993[9]) can pave the way for tumorigenesis and cancer progression (Rajabi and Mousa, 2017[25]; Mohajeri et al., 2017[22]). In support of this hypothesis we also found association between the *2-2* genotype/phenotype with T category/ grade of the tumor in NMIBC patients. Pirincci et al. (2012[24]) also represented the association between Hp levels with size, extent of distant metastasis, cancer cell migration and T category/ grade of a tumor. 

Therefore, the possible mechanisms that through them *2-2* genotype/phenotype can induce bladder cancer development are as follows: lowering the antioxidant capacity, accumulation of free radicals in the presence of free hemoglobin, enhancement of iron accumulation and facilitating cancer cell migration and angiogenesis. 

It should be mentioned that although the present study was conducted with relatively small sample size but we recruited new cases with NMIBC and also the ‎2D-GE/MS was used ‎for phenotyping which is a more precise and sensitive method. However, for further clarification new studies with larger sample size, as well as, studies on other types of bladder cancer are required. 

In conclusion, the present study implied a strong association between the Hp phenotypes/genotypes with NMIBC. It was found that the *2-2 *genotype/phenotype could be considered as risk factors as well as could be useful biomarkers in diagnosis of new bladder cancer cases. This study introduced Hp genotyping as a possible cost-effective and precise method for prognosis of individuals at the risk of NMIBC.

## Acknowledgements

This work was supported by Drug Applied Research Center, Tabriz University of Medical Sciences, Tabriz, Iran. The authors thank Drug Applied Research Center, Tabriz University of Medical Sciences, Hasheminejad Kidney Center (HKC), and all the patients and healthy individuals who participated in this study.

## Funding

This study was funded by Drug Applied Research Center, Tabriz University of Medical Sciences, Tabriz, Iran [grant number 7683/23].

## Conflict of interest

The authors declare that they have no conflict of interest.

## Figures and Tables

**Table 1:**
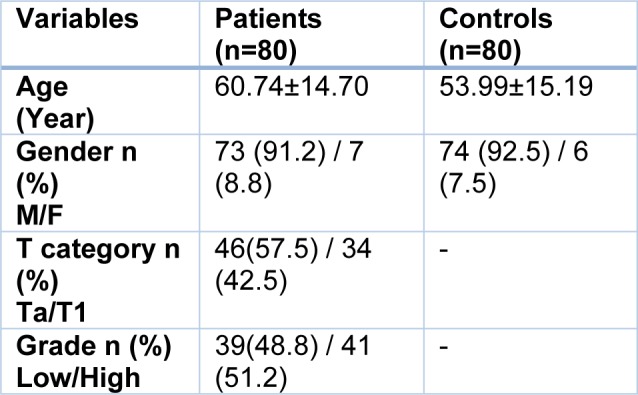
Demographic and clinicopathological variables in non-muscle invasive bladder cancer patients and healthy controls

**Table 2:**
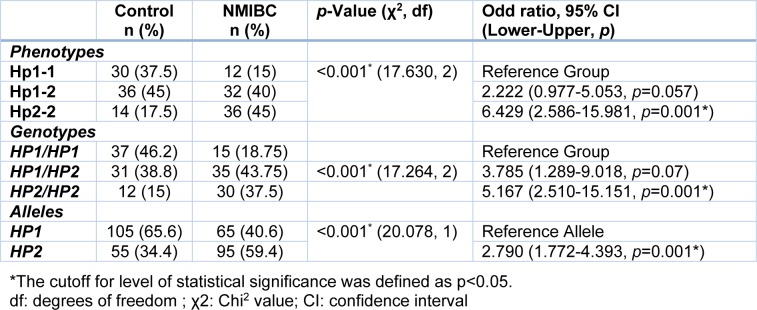
The frequency of haptoglobin phenotypes and genotypes in non-muscle invasive bladder cancer (NMIBC) patients (n=80) and healthy controls (n=80) using Chi^2^ test

**Table 3:**
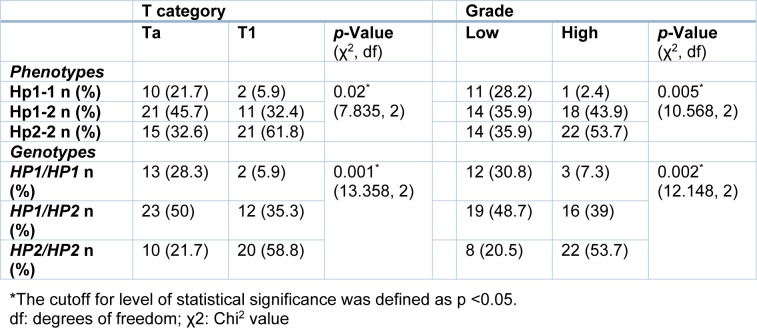
T category and grade of tumor in patients with transitional cell carcinomas regarding phenotypes and genotypes of haptoglobin using Chi^2^ test
